# The Correlation between FSTL1 Expression and Airway Remodeling in Asthmatics

**DOI:** 10.1155/2017/7918472

**Published:** 2017-08-03

**Authors:** Yahui Liu, Tian Liu, Jinxiang Wu, Tao Li, Xingai Jiao, Haiqing Zhang, Jiping Zhao, Junfei Wang, Lin Liu, Liuzhao Cao, Shuo Li, Jiawei Xu, Jianfeng Xu, Xiaohui Ma, Lei Yang, Liang Dong

**Affiliations:** ^1^Department of Pulmonary Medicine, Qilu Hospital of Shandong University, Jinan 250012, China; ^2^Department of Pulmonary Medicine-Bronchoscopy Room, Qilu Hospital of Shandong University, Jinan 250012, China

## Abstract

**Background:**

Asthma is characterized by airway remodeling. Follistatin-like protein 1 (FSTL1) is an extracellular glycoprotein. Recent studies suggest that FSTL1 may participate in the pathogenesis of asthma.

**Objectives:**

To analyze the association between FSTL1 and some parameters and inspect the role of FSTL1 in asthma.

**Methods:**

We examined FSTL1 levels in 32 asthmatics and 25 controls. All subjects enrolled had routine blood tests, spirometry, and impulse oscillometry performed. Additionally, 15 of the 32 asthmatics underwent fibre optic bronchoscopy. Spearman rank analysis was performed to detect the correlation between FSTL1 and other parameters.

**Results:**

Plasma FSTL1 levels were higher in asthmatics (130.762 ± 46.029 ng/mL) than in controls (95.408 ± 33.938 ng/mL) (*p* = 0.009). Plasma FSTL1 levels were associated with fibrosis levels around the airways (rs = 0.529, *p* = 0.043) and *α*-smooth muscle actin (*α*-SMA) (rs = 0.554, *p* = 0.032). FSTL1 levels in bronchoalveolar lavage fluid were associated with collagen I (rs = 0.536, *p* = 0.040), *α*-SMA (rs = 0.561, *p* = 0.029), fibrosis levels (rs = 0.779, *p* = 0.001), and the thickness of the airway reticular basement membrane (RBM) (rs = 0.660, *p* = 0.007).

**Conclusions:**

FSTL1 levels in asthmatics were linked with increased smooth muscle mass and thickened RBM. FSTL1 may contribute to airway remodeling in asthmatics.

## 1. Introduction

Asthma is a chronic respiratory disease usually characterized by chronic airway inflammation, airway remodeling, and airway hyper responsiveness [1]. Airway remodeling refers to the structural changes in the airway including, but not limited to, the airway smooth muscle, airway epithelia, blood vessels, and extracellular matrix [2]. Features of airway remodeling in asthma include an increase of airway smooth muscle mass, epithelial injury, epithelial cell hyperplasia, goblet cell metaplasia, reticular basement membrane (RBM) thickening, and angiogenesis [2]. Previous studies have indicated that these structural changes contribute to asthma persistence, airflow obstruction, lung function decline, and clinical severity [3, 4]. The mechanisms of airway remodeling, however, are still unclear. There is evidence to show that multiple cytokines, chemokines, transcription factors, and growth factors released from inflammatory and structural cells in the airway are involved in airway remodeling, such as transforming growth factor beta (TGF-*β*), vascular endothelial growth factor (VEGF), Th2 cytokines (IL-5, IL-9, and IL-13), and epithelial-derived nuclear factor kappa-light-chain-enhancer of activated B cells (NF-*κ*B) [5].

Follistatin-like protein 1 (FSTL1), also known as transforming growth factor (TGF) *β*1-stimulated clone 36 (TSC-36) or follistatin-related protein (FRP), is a secreted glycoprotein of 308 amino acids [6, 7]. Although the function of FSTL1 is not completely understood, it has been shown that FSTL1 plays a key role in tumor bone metastasis [8, 9], chronic pain hypersensitivity [10], inflammation and insulin resistance in obesity [11], the regulation of erythropoiesis [12], physical development [13–16], and somatic sensation by binding to Na^+^,K^+^-ATPase [17]. More importantly, many studies have demonstrated that FSTL1 upregulates proinflammatory mediators in the pathology of arthritis and serum levels of FSTL1 correlating with severity of arthritis [18–20]. However, FSTL1 may offer protection of the heart as it is essential for the acute repair of the infarcted myocardium [21] and can antagonize myocyte hypertrophic growth and the loss of ventricular performance in response to pressure overload [22]. In murine and porcine models of ischemia/reperfusion, FSTL1 prevents myocardial ischemia/reperfusion injury [23].

Several studies have shown that FSTL1 may play an important role in the respiratory system. FSTL1 modulates lung development, cartilage formation, and alveolar maturation [13, 24, 25]. Knockout of FSTL1 in mice is embryonic-lethal, and these mice display multiple developmental abnormalities of the respiratory and skeletal system [13]. Additionally, FSTL1 has a role in cellular proliferation and apoptosis in lung cancer [26]. Lung injury can induce the production of FSTL1 which can promote the accumulation of myofibroblasts and subsequently lead to fibrosis [27]. Miller et al. demonstrated that FSTL1 is highly expressed by macrophages in the lungs of people with severe asthma and that the FSTL1/oncostatin M pathway may promote airway remodeling in severe asthma [28]. Here, we aimed to compare the concentration of FSTL1 in plasma and bronchoalveolar lavage fluid (BALF) in patients with and without asthma. We then aimed to analyze the association between FSTL1 and a series of clinical parameters to help identify mechanisms behind the pathology of airway remodeling in asthma.

## 2. Materials and Methods

### 2.1. Study Population

In our study, 32 subjects who visited the Respiratory Clinic of Qilu Hospital, Shandong University (Jinan, Shandong, China), and were diagnosed with asthma according to the Global Initiative for Asthma (2015 edition) were enrolled. All patients were required to meet at least one of the following eligibility criteria: a history of wheezing, cough, chest tightness, and/or dyspnea; having a positive bronchodilator response of 12% and 200 mL increase in forced expiratory volume in one second (FEV1); and/or day to day airflow variability. All patients were treatment naive prior to enrolment. We also recruited 25 subjects who visited our hospital for a routine medical checkup but did not have any respiratory condition as nonasthmatic controls.

All subjects (asthmatics and controls) had no history of primary cardiomyopathy, secondary cardiomyopathy, congenital heart disease, renal failure, autoimmune diseases, pulmonary fibrosis, cardiac surgery, and coronary artery therapy as these conditions may influence the plasma levels of FSTL1. Patients who had any symptoms of respiratory infection in the 2 weeks prior were also excluded.

The research protocol was approved by the Ethics Review Committee for Human Studies at Qilu Hospital, Shandong University, and all the subjects provided written informed consent.

### 2.2. Routine Blood and Pulmonary Function Tests

All subjects had routine blood, pulmonary function tests (spirometry), and impulse oscillometry (IOS) at Qilu Hospital of Shandong University. Spirometry was conducted using MasterScreen™ Pneumo spirometer (Jaeger Co., Hoechberg, Germany) and carried out according to ATS/ERS criteria [29]. IOS was conducted on Jaeger MasterScreen IOS (Jaeger Co.), and tests were performed prior to spirometry. Trials lasted approximately 40 seconds and contained 200–250 separate impulses.

### 2.3. Bronchoscopy

Flexible bronchoscopy was performed in 15 of the 32 asthmatics, and this operation was approved by local institutional review boards. Written informed consent was obtained from these patients. Postmortem human lungs from donors without history of asthma (*n* = 5) were also obtained from the Qilu Hospital Cadaver Donating Center in a protocol approved by the Ethics Review Committee for Human Studies at Qilu Hospital, Shandong University. Biopsies from these postmortem samples acted as controls.

All bronchoscopies were performed under topical anesthesia using the Olympus BF-UC260F-0L8 bronchofibrevideoscope. Bronchoalveolar lavage (BAL) was performed using 3 aliquots of 1 mL/kg 0.9% sterile saline (to a maximum of 40 mL per aliquot) instilled into the right middle lobe or an area of radiographically defined abnormality and the returns pooled. Up to 3 biopsies were taken from each subject. The mean number of biopsies per subject was 1.47.

### 2.4. FSTL1 Concentration in Plasma and BALF

FSTL1 levels in plasma and BALF were measured by a commercially available enzyme-linked immunosorbent assay (ELISA) kit from CUSABIO, MD, USA. The kit was used according to the manufacturer's instructions with the standard curve generated using Curve Expert 1.3. The detection range was 12.5 ng/mL–800 ng/mL. The intra-assay and interassay coefficients of variation were less than 8% and 10%, respectively.

### 2.5. Staining of Bronchial Biopsies and Quantification of Histological Images

Bronchoscopy was performed in 15 of the 32 asthmatics. For normal control samples, postmortem nonasthmatic human lungs (*n* = 5) were obtained from the Qilu Hospital Cadaver Donating Center under a protocol approved by the Ethics Review Committee for Human Studies at Qilu Hospital, Shandong University. And they did not suffer from asthma according to their antemortem history. All specimens were obtained by fine aspiration followed by immersing the samples immediately into a 4% solution of formaldehyde. After 24 hours, samples were embedded in paraffin and stored at 4°C before cutting them into 5 *μ*m slices.

Bronchial biopsies and postmortem human lung samples were stained with hematoxylin and eosin, periodic acid silver methenamine, and Sirius red.

Immunohistochemisty (IHC) was performed on 5 *μ*m thick paraffin-embedded sections of human lung samples. Slides were deparaffinized and pretreated with 1 mmol/L EDTA and heat-mediated antigen retrieval solution (Solarbio) in a microwave oven. The antigen was blocked in 20% normal fetal bovine serum (ZSGB, Beijing, China). Sections were incubated with rabbit anti-FSTL1 antibody (Abcam, Cambridge, UK) at 1 : 100 dilution, rabbit anti-collagen I (Bioss, MA, USA) at 1 : 100 dilution, and mouse anti-*α*-SMA (BOSTER, CA, USA) at 1 : 100 dilution. Sections were then incubated with peroxidase-conjugated goat anti-rabbit IgG (ZSGB, Beijing, China) at 1 : 500 dilution and peroxidase-conjugated goat anti-mouse IgG (ZSGB) at 1 : 500 dilution, followed by addition of streptavidin-HRP conjugate and substrate-chromogen mixture. Finally, all slides were counterstained with hematoxylin.

Quantification of staining on histological images was performed using ImageJ at ×400 magnification. Images were corrected for white balance before color deconvolution. We selected the all the noncontiguous regions of interest (ROI) to calculate the integral optical density (IOD). By dividing IOD by the area, we can get the average optical density (AOD). For the expression of each kind of protein (*FSTL1*, *α-SMA*, and *collagen I*), three sections of IHC were used to calculate the AOD per subject. Five random areas of each section were measured in each PSAM staining slides to determine RBM thickness and fibrosis levels. Results are the mean of three slides per patient in *μ*m.

### 2.6. Follow-Up of Asthmatic Patients

After one month, more than three quarters of our enrolled asthmatics returned to our clinic for follow-up, of whom 17 subjects were well controlled and did not suffer from any other respiratory complications, such as pneumonia. Four months after their initial visit, 10 subjects returned for follow-up. Blood samples were collected for routine blood, and spirometry was performed. Subjects received treatment (inhaled corticosteroids and long-acting *β*-agonist combination therapy and/or oral leukotriene receptor antagonists) for their asthma after their first visit.

### 2.7. Statistical Analysis

All data are presented as mean ± standard deviation (SD). Data was first tested for normality using the Shapiro-Wilk test before analysis with the appropriate ANOVA test. Plasma levels of FSTL1 were logarithmically transformed (log_10_) because of their skewed distribution. FSTL1 and other parameters were examined by Spearman rank correlation analysis. A value of *p* < 0.05 was considered as statistically significant. All analyses were performed using SPSS (version 20) (IBM, NY, USA). The quantitative analysis of figures of IHC, PASM staining, and Sirius red staining was performed using ImageJ.

## 3. Results

### 3.1. Characteristics across Controls and Asthmatics

32 patients with asthma completed our study, among whom 11 were male (34.4%). Of the 25 controls, 10 were male (40.0%). Characteristics of the patients are described in Table 1. There was no difference between the groups in age, gender, or BMI.

**Figure 1:**
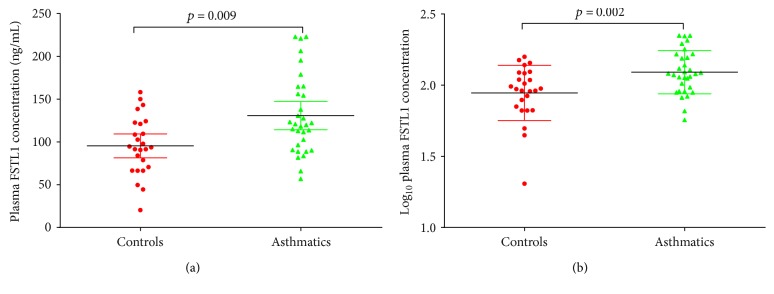
Plasma FSTL1 levels in controls and asthmatics. (a) Plasma FSTL1 levels in controls and asthmatics. (b) log_10_FSTL1 levels in controls and asthmatics from plasma. Data is represented as mean ± SD.

The concentration (*p* = 0.006) and percentage (*p* = 0.008) of eosinophils in the blood, respiratory system impedance (Zrs) (*p* < 0.001), resonant frequency (Fres) (*p* < 0.001), resistance at 5 Hz (R5) (*p* < 0.001), resistance at 20 Hz (R20) (*p* < 0.001), R5 minus R20 (R5-R20) (*p* < 0.001), and reactance at 5 Hz (X5) (*p* < 0.001) were all higher in the asthma group compared to the control group. Forced expiratory volume in one second (FEV_1_) and FEV_1_/forced vital capacity (FVC) were higher in controls than in asthmatics (*p* < 0.001 and *p* < 0.001, resp.) (Table 1). Of note, plasma concentrations of FSTL1 in the asthma group (130.762 ± 46.029 ng/mL) were significantly elevated compared to controls (95.408 ± 33.938 ng/mL) (*p* = 0.009) (Figure 1).

### 3.2. FSTL1 and Lung Function and IOS

**Figure 2:**
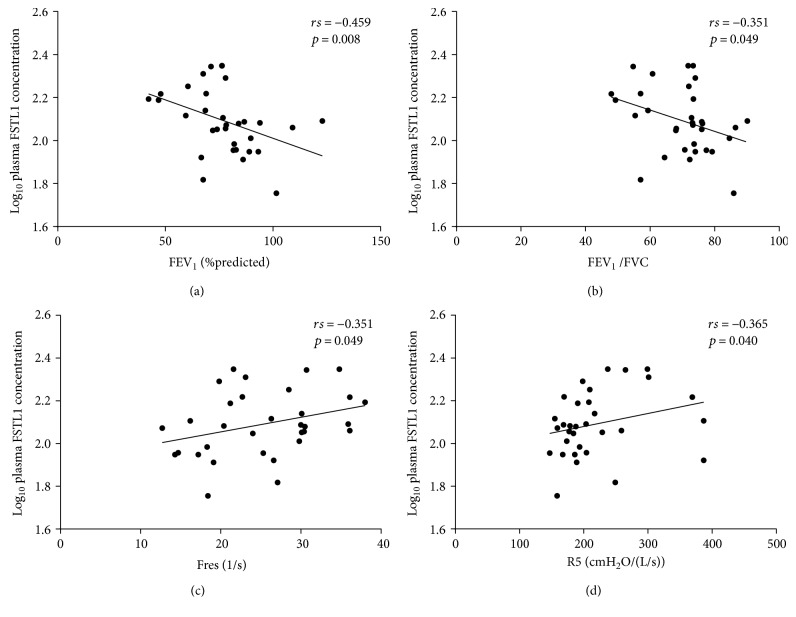
Correlation between plasma log_10_FSTL1 levels and spirometric and IOS parameters. Correlations between plasma log_10_FSTL1 levels and (a) FEV_1_ (%predicted), (b) FEV_1_/FVC, (c) Fres, and (d) R5. Correlations were determined by Spearman rank correlation analysis.

There was a positive correlation between log_10_FSTL1 levels in plasma and Fres (rs = 0.351, *p* = 0.049) as well as R5 (rs = 0.365, *p* = 0.040) (Table 2 and Figure 2). Plasma FSTL1 levels were inversely related to FEV_1_ (rs = −0.459, *p* = 0.008) and FEV_1_/FVC (rs = −0.351, *p* = 0.049) (Table 2 and Figure 2).

### 3.3. FSTL1 Levels in BALF with Asthma

**Figure 3:**
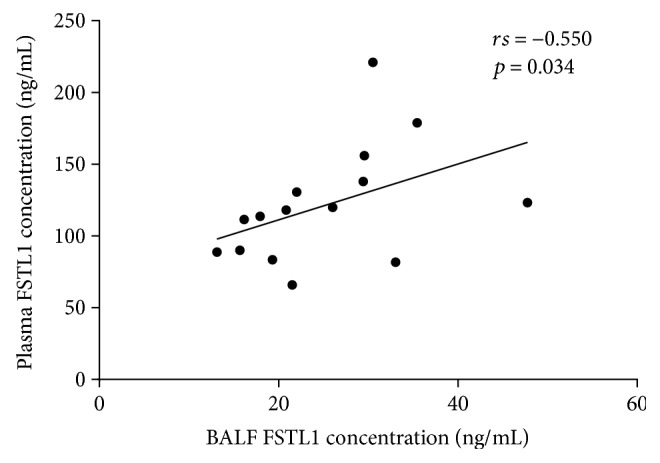
Correlation between FSTL1 levels in plasma and BALF. Correlation was determined by Spearman rank correlation analysis.

BALF was collected from 15 asthmatics when they underwent fibre optic bronchoscopy before sampling. FSTL1 levels in BALF positively correlated with plasma FSTL1 concentration (rs = 0.550, *p* = 0.034) (Figure 3).

### 3.4. Staining of the Bronchial Biopsies

**Figure 4:**
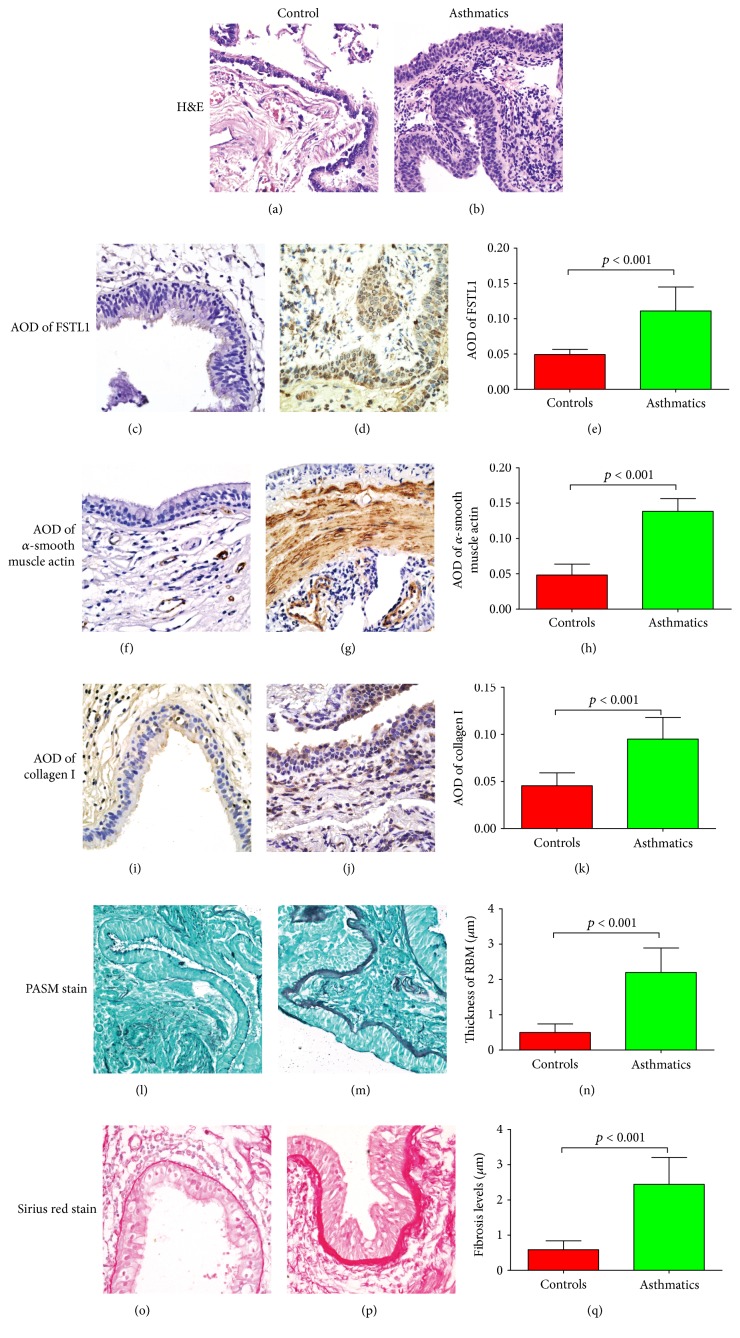
Staining of bronchial biopsies of both controls (postmortem samples) and asthmatics and quantitative analysis. H&E stains for (a) controls and (b) asthmatics. Expression of FSTL1 by IHC in (c) controls and (d) asthmatics and (e) quantification of FSTL1 staining. Expression of *α*-smooth muscle actin by IHC in (f) controls and (g) asthmatics and (h) quantification of *α*-smooth muscle actin staining. Expression of collagen I by IHC in (i) controls and (j) asthmatics and (k) quantification of collagen I staining. Expression of basement membrane thickness by PASM staining in (l) controls and (m) asthmatics and (n) quantification of basement membrane staining. Expression of fibrosis levels by Sirius red staining in (o) controls and (p) asthmatics and (q) quantification of fibrosis levels. Original magnification ×400. Data is represented as mean ± SD.

At high magnification, H&E stains of asthmatic sections showed that the epithelium was mostly intact but infiltrated by inflammatory cells (Figure 4(b)). Epithelial cells were also longer than normal in asthmatic airways (Figures 4(a) and 4(b)). In normal controls, the reticular basement membrane contained few inflammatory cells (Figure 4(a)). Sloughed cells found in control sections were partly the result of postmortem changes and pathological changes due to diseases other than asthma (Figure 4(a)).

IHC staining demonstrated higher FSTL1 expression in asthmatic sections than in controls (*p* < 0.001) (Figures 4(c), 4(d), and 4(e)). FSTL1 expression in control sections was mostly in the epithelia and nonspecific staining on the top of the ciliated cells (Figure 4(c)). However, in asthmatic sections, FSTL1 was expressed not only in mesenchymal cells but also in epithelia (Figure 4(d)).

Similarly, expression of *α*-SMA and collagen I were higher in asthmatics than in nonasthmatic controls (*p* < 0.001 and *p* < 0.001, resp.) (Figures 4(f), 4(g), 4(h), 4(i), 4(j), and 4(k)). IHC staining showed that *α*-SMA was expressed in the vascular endothelial cells in controls. However, *α*-SMA was highly expressed in both vascular endothelial cells and the submucosa in asthmatics, demonstrating increased smooth muscle mass in asthmatics (Figures 4(f) and 4(g)). In controls, mesenchymal cells expressed collagen I (Figure 4(i)). However, in asthmatic sections, epithelial cells also stained positive for collagen I (Figure 4(j)).

PASM staining of the airway basement membrane was greater in asthmatics compared to that in controls (Figures 4(l) and 4(m)) and was determined quantitatively to be thicker in asthmatic sections (*p* < 0.001) (Figure 4(n)).

Sirius red staining for fibrosis around the airways showed that there was increased collagen deposition around the airway in asthmatics compared with that in the controls (Figures 4(o), 4(p), and 4(q)).

### 3.5. Correlation between FSTL1 and Airway Remodeling

**Figure 5:**
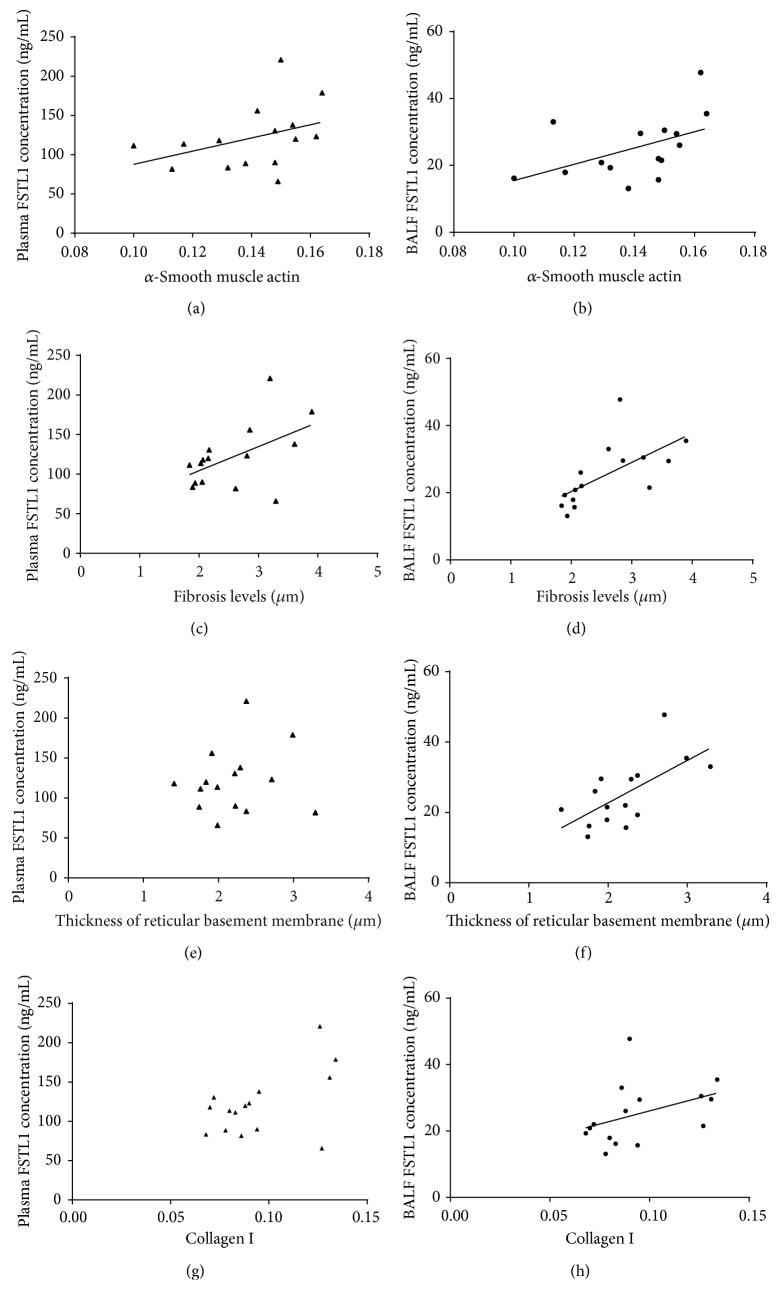
Relationships between FSTL1 levels in plasma and BALF and airway remodeling. Correlation between *α*-smooth muscle actin and (a) plasma FSTL1 concentration and (b) BALF FSTL1 concentration. Correlation between fibrosis levels and (c) plasma FSTL1 concentration and (d) BALF FSTL1 concentration. Correlation between basement membrane thickness and (e) plasma FSTL1 concentration and (f) BALF FSTL1 concentration. Correlation between collagen I levels and (a) plasma FSTL1 concentration and (b) BALF FSTL1 concentration. Correlation was determined by Spearman rank correlation analysis.

Bronchial biopsies of 15 asthmatics were collected, and Spearman rank correlation analysis demonstrated that plasma FSTL1 levels were correlated positively with the expression of *α*-SMA in bronchial biopsies (rs = 0.554, *p* = 0.032) (Figure 5(a)) and fibrosis levels around the airways (rs = 0.529, *p* = 0.043) (Figure 5(c)). Furthermore, the levels of BALF FSTL1 correlated with the expression of *α*-SMA (rs = 0.561, *p* = 0.029) (Figure 5(b)), fibrosis levels around the airways (rs = 0.779, *p* = 0.001) (Figure 5(d)), thickness of basement membrane (rs = 0.660, *p* = 0.007) (Figure 5(f)), and collagen I (rs = 0.536, *p* = 0.040) (Figure 5(h)).

### 3.6. Changes before and after Treatment

**Figure 6:**
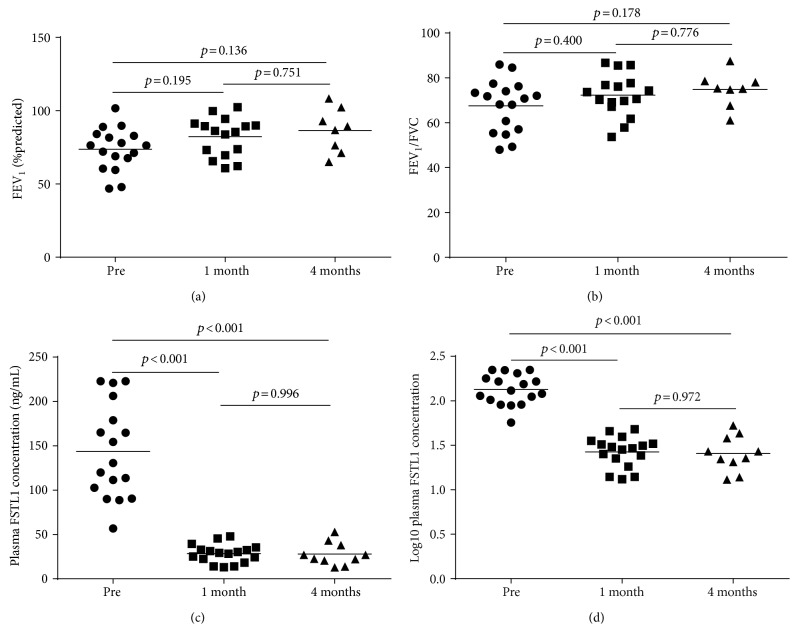
The changes of spirometry and FSTL1 levels before and after treatment. (a) FEV_1_ (%predicted) before treatment, 1 month posttreatment, and 4 months posttreatment. (b) FEV_1_/FVC before treatment, 1 month posttreatment, and 4 months posttreatment. (c) Plasma FSTL1 concentration before treatment, 1 month posttreatment, and 3 months posttreatment. (d) Log_10_ plasma FSTL1 concentrations before treatment, 1 month posttreatment, and 3 months posttreatment. Multiple comparison was performed by Games-Howell test. Line indicates mean.

Figure 6 shows the changes of observed values before treatment, one month after treatment, and four months after treatment. During the period of follow-up, the concentration of FSTL1 in plasma decreased over time from pretreatment (143.519 ± 53.194 ng/mL) to one month after treatment (28.447 ± 10.325 ng/mL) (*p* < 0.001) (Figures 6(c) and 6(d)). However, there were no changes between plasma FSTL1 levels between one-month follow-up (28.447 ± 10.325 ng/mL) and four months (28.023 ± 12.891 ng/mL) after treatment (*p* = 0.996) (Figures 6(c) and 6(d)). With regard to FEV_1_ and FEV_1_/FVC, there was a general increasing trend; however, the changes were not statistically significant (Figures 6(a) and 6(b)).

## 4. Discussion

In this study, we demonstrated that the levels of FSTL1 in the plasma were elevated in asthmatics versus those in age- and gender-matched controls. For asthmatics, there was a correlation between plasma FSTL1 levels and FEV_1_, FEV_1_/FVC, Fres, R5, and FSTL1 levels in BALF. IHC staining of human bronchial biopsies and quantitative analysis demonstrated that FSTL1 was expressed more highly in biopsies of asthmatics than in those of controls. Furthermore, plasma FSTL1 levels correlated with the expression of *α*-SMA in bronchial biopsies and fibrosis levels around the airways. BALF FSTL1 concentrations were correlated with the expression of collagen I and *α*-SMA, the thickness of reticular basement membrane, and fibrosis levels around the airways. We found that the concentration of plasma FSTL1 decreased significantly after 1 month of treatment with inhaled corticosteroids and long-acting *β*-agonists combination therapy and/or oral leukotriene receptor antagonists.

Our study demonstrated that there was a relationship between FSTL1 levels in plasma and BALF, with concentrations being higher in plasma than those in BALF. We speculate that during the bronchoscopy procedure where BALF was collected, the routinely used saline diluted the FSTL1 concentration. The relationship between FSTL1 levels in plasma and the parameters of airway remodeling was weaker than the relationship between FSTL1 levels in BALF and parameters of airway remodeling, suggesting its importance in the remodeling process. Although we set strict inclusive and exclusive criteria, subjects enrolled in this study may have had some other underlying conditions, which could influence the concentration of FSTL1, especially plasma concentration. Despite this, FSTL1 levels in plasma did relate to airway remodeling of parameters to a certain degree since they were linked with expression of *α*-SMA in bronchial biopsies and fibrosis levels around the airways, suggesting some utility of its use as a blood biomarker.

The increased expression of FSTL1 in biopsies was accompanied by more expression of *α*-SMA collagen I and a thicker reticular basement membrane, which are all the hallmarks of airway remodeling in asthma [2, 30]. The thickened reticular basement membrane has been reported to be due to the deposition of collagens I and III, tenascin, fibronectin, and immunoglobulin (Ig), which can lead to subepithelial fibrosis [31]. The relationship between hallmarks of airway remodeling and FSTL1 levels in BALF demonstrated that FSTL1 levels could reflect the degree of reticular basement membrane thickening and smooth muscle mass increase. Our study demonstrated that plasma FSTL1 levels were correlated with fibrosis levels around airways and smooth muscle mass increase, but not the expression of the collagen I and basement membrane thickness. Considering that plasma FSTL1 levels fluctuate and decrease with asthma treatment and the specificity of BALF FSTL1 concentrations with remodeling parameters, we speculate that FSTL1 may play an important role in airway remodeling.

Moreover, after treatment for asthma, the levels of FSTL1 in plasma declined, but due to moral and ethical reasons, we were unable to assess airway remodeling with additional bronchoscopy procedures. Inhaled corticosteroids and/or oral leukotriene receptor antagonists could decrease the expression of FSTL1 in plasma directly or indirectly. Given our findings that plasma and BALF FSTL1 concentrations correlate, we speculate that with treatment, BALF FSTL1 levels would also be reduced with treatment. Whether airway remodeling in asthma is halted or slowed or if the decline of FSTL1 is linked with the improvement of pulmonary function, testing is still not clear and requires further investigation.

Since elevated levels of FSTL1 coexist with airway remodeling in asthma, we wonder whether FSTL1 participates in airway remodeling as a mediator or whether FSTL1 levels increase following airway remodeling as a kind of maladaption. For the time being, the mechanisms of airway remodeling in asthma are still unclear. Previous studies demonstrated that decreased levels of FSTL1 in heterozygous FSTL1^+/−^-deficient mice attenuated bleomycin-induced pulmonary fibrosis through a TGF-*β*-dependent pathway [27]. Miller et al. found that administration of FSTL1 to WT mice induces airway remodeling in a mouse model of asthma [28]. Investigation of molecular mechanisms was outside the scope of our study, but taking previous work in the literature into consideration, we believe that FSTL1 is likely to promote airway remodeling in asthma. However, the exact mechanisms by which this occurs still need to be elucidated.

In general, FSTL1 is secreted by nonhematopoietic cells such as cells of the mesenchymal lineage, such as fibroblasts [32]. Our study found that the airway epithelia also expressed FSTL1. However, the role of FSTL1 expression in epithelial cell is currently unknown and further studies are required to determine its role in airway remodeling.

Asthma is a heterogeneous disease, and according to Masoli et al., the global burden of asthma accounts for about 1% of disability-adjusted life years of all diseases around the world [33]. More effective therapies are needed and one of the hurdles to developing therapeutics is the lack of complete understanding of the mechanisms underlying the pathogenesis of this disease. Our findings here add new insights into the understanding of asthma and indicate a potential novel role of FSTL1 in asthma.

## 5. Conclusion

In summary, our study demonstrated that the levels of plasma FSTL1 were elevated in asthmatics and the levels of FSTL1 in plasma and BALF were positively correlated in people with asthma. There was an association between FSTL1 and airway remodeling in asthmatics without treatment suggesting that it may promote airway remodeling in people with asthma.

## Figures and Tables

**Table 1:** Patient characteristics.

	Controls	Asthmatics	*p* value
*n*	25	32	
Age (years)	40.84 ± 18.665	42.31 ± 15.422	0.746^a^
Gender, male (%)	10 (40.000)	11 (34.375)	0.662^b^
BMI (kg/m^2^)	24.748 ± 2.976	25.500 ± 4.537	0.477^a^
WBC# (×10^9^/L)	6.251 ± 2.008^‡^	7.460 ± 2.787	0.092^a^
neu# (×10^9^/L)	3.935 ± 1.998^‡^	4.609 ± 2.829	0.348^a^
eos# (×10^9^/L)	0.100 ± 0.105^‡^	0.473 ± 0.706	**0.006** ^c^
neu%	61.148 ± 14.608^‡^	58.813 ± 15.927	0.592^a^
eos%	1.652 ± 1.687^‡^	5.938 ± 8.378	**0.008** ^c^
log_10_ (FSTL1)	1.945 ± 0.194	2.091 ± 0.152	**0.002** ^a^
FSTL_1_ (ng/mL)	95.408 ± 33.938	130.762 ± 46.029	**0.009** ^d^
FEV_1_ (%predicted)	99.245 ± 10.236	77.502 ± 16.989	**<0.001** ^c^
FEV_1_/FVC	84.787 ± 3.087	70.261 ± 10.521	**<0.001** ^c^
Zrs (Ω)	104.252 ± 27.344	271.991 ± 91.064	**<0.001** ^c^
Fres (1/s)	11.436 ± 2.754	25.313 ± 7.088	**<0.001** ^c^
R5 (cmH_2_O/(L/s))	101.052 ± 27.009	216.334 ± 67.657	**<0.001** ^c^
R20 (cmH_2_O/(L/s))	96.620 ± 27.588	157.634 ± 24.796	**<0.001** ^a^
R5-R20 (cmH_2_O/(L/s))	10.208 ± 7.351	63.944 ± 60.949	**<0.001** ^c^
X5 (cmH_2_O/(L/s))	0.599 ± 0.451	4.190 ± 2.570	**<0.001** ^c^

Data are presented as mean ± SD; the values of WBC#, neu#, eos#, neu%, and eos% were in blood. The values of FSTL1 were in plasma; BMI: body mass index; WBC#: white blood cell count; neu#: neutrophil count; eos#: eosinophil count; neu%: neutrophil percentage; eos%: eosinophil percentage; FEV_1_: forced expiratory volume in one second; FEV_1_/FVC: forced expiratory volume in one second/forced vital capacity; Zrs: respiratory system impedance; Fres: resonant frequency; R5: resistance at 5 Hz; R20: resistance at 20 Hz; X5: reactance at 5 Hz; R5-R20: R5 was subtracted by R20; ^a^two-tailed independent sample *t*-test; ^b^Pearson chi-square test; ^c^separate variance estimation *t*-test; ^d^Mann–Whitney *U*; ^‡^*n* = 21.

**Table 2:** Spearman rank correlation analysis to identify factors associated with log_10_FSTL1 in asthmatics.

	Correlation coefficient	*p* value
Age (years)	−0.241	0.185
BMI (kg/m^2^)	0.081	0.658
eos# (×10^9^/L)	0.309	0.086
eos%	0.253	0.163
FEV_1_ (%predicted)	−0.459	**0.008**
FEV_1_/FVC	−0.351	**0.049**
Zrs (Ω)	0.157	0.389
Fres (1/s)	0.351	**0.049**
R5 (cmH_2_O/(L/s))	0.365	**0.040**
R20 (cmH_2_O/(L/s))	0.247	0.173
R5-R20 (cmH_2_O/(L/s))	0.263	0.147
X5 (cmH_2_O/(L/s))	0.162	0.375

BMI: body mass index; eos#: eosinophil count; eos%: eosinophil percentage; FEV_1_: forced expiratory volume in one second; FEV_1_/FVC: forced expiratory volume in one second/forced vital capacity; Zrs: respiratory system impedance; Fres: resonant frequency; R5: resistance at 5 Hz; R20: resistance at 20 Hz; X5: reactance at 5 Hz; R5-R20: R5 was subtracted by R20.

## Abbreviations

FSTL1: Follistatin-like protein 1
BALF: Bronchoalveolar lavage fluid
IOS: Impulse oscillometry
ELISA: Enzyme-linked immunosorbent assay
BMI: Body mass index
WBC#: White blood cell count
Eos#: Eosinophil count
Eos%: Eosinophil percentage
neu#: Neutrophil count
neu%: Neutrophil percentage
FEV_1_: Forced expiratory volume in one second
FEV_1_/FVC: Forced expiratory volume in one second/forced vital capacity
Zrs: Respiratory system impedance
Fres: Resonant frequency
R5: Resistance at 5 Hz
R20: Resistance at 20 Hz
X5: Reactance at 5 Hz
R5-R20: R5 minus R20
rs: Spearman rank correlation coefficient.
